# Novel Cytotoxic Phenanthro-triazine-3-thiol Derivatives as Potential DNA Intercalators and Bcl-2 Inhibitors

**DOI:** 10.22037/ijpr.2020.113902.14553

**Published:** 2021

**Authors:** Mehdi Khoshneviszadeh, Omidreza Firuzi, Malihe Aminsafaee, Masoud Kashefizadeh, Sara Ranjbar, Zahra Rezaei, Hossein Sadeghpour, Farshid Zargari, Ramin Miri, Najmeh Edraki

**Affiliations:** a *Medicinal and Natural Products Chemistry Research Center, Shiraz University of Medical Sciences, Shiraz, Iran. *; b *Department of Medicinal Chemistry, Faculty of Pharmacy, Shiraz University of Medical Sciences, Shiraz, Iran. *; c *Pharmaceutical Sciences Research Center, Shiraz University of Medical Sciences, Shiraz, Iran.*

**Keywords:** Triazine, Phenanthrene, Cancer, Antiproliferative effect, Molecular docking

## Abstract

Novel phenanthro-triazine-3-thiol derivatives were designed as potential DNA intercalators and Bcl-2 inhibitors. After being synthesized, the compounds were evaluated for their cytotoxic activity against MOLT-4 (human acute lymphoblastic leukemia) and MCF-7 (human breast adenocarcinoma) cells by MTT assay. **P1** (bearing hydrogen substitution) was the most potent derivative against MOLT-4 with an IC_50 _value of 7.1 ± 1.1 μM, whereas **P11 **(bearing phenyl substitution) demonstrated considerable cytotoxicity against MCF-7 with an IC_50 _value of 15.4 ± 2.9 μM. Compounds **P7**, **P8**, **P14** and **P15 **exhibited moderate cytotoxic effects. Furthermore, to confirm the potential DNA intercalation and Bcl-2 inhibitory activities of phenanthro-triazine scaffolds, molecular docking analysis was performed. Molecular docking studies indicated that these compounds not only bind to DNA by intercalation mainly through stacking interactions but also are well accommodated in the active site of Bcl-2. Therefore, **P1** and **P11** having phenanthro-triazine-3-thiol scaffold could be presented as cytotoxic agents with dual DNA intercalation and Bcl-2 inhibitory activities.

## Introduction

Despite the availability of various cancer therapeutic agents, we continue to face complications and a lack of efficacy of available drugs. The Discovery of potential novel chemotherapeutics with improved efficacy and fewer side effects is an enormous challenge (1, 2). 

**Figure 1:**
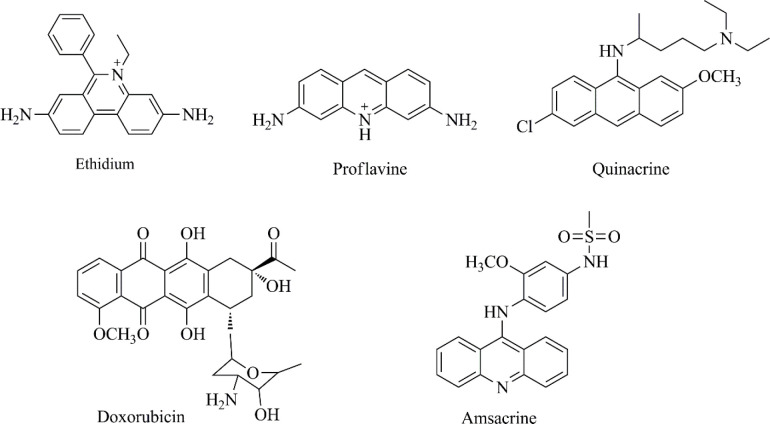
Cytotoxic organic intercalators bearing phenanthridine (ethidium), acridine (proflavine and amsacrine), anthracene (quinacrine) and anthraquinone (doxorubicin) skeletons

DNA is still one of the most important targets for anticancer drug discovery (3, 4). DNA intercalating agents are likely to reversibly bind DNA and interfere with the essential functions of the cancer cells, such as transcription and replication (5-7). DNA organic intercalators mostly contain planar polycyclic aromatic rings (such as anthracene, acridine, anthraquinone and phenanthridine cores) helping the compound accommodate between base pairs, and also possess some polar functions providing hydrogen bond interactions with nucleic acids (7-9). Structures of some antitumor agents as DNA intercalators are depicted in Figure 1.

Additionally, B-cell lymphoma-2 (Bcl-2) family proteins are important regulators of apoptosis which are generally overexpressed in many cancer cells. Therefore, another trend for the treatment of cancer is finding inhibitors targeting the Bcl-2 proteins. The Bcl-2 family is divided into three groups based on their primary function 1) anti-apoptotic proteins (Bcl-2, Bcl-xL, Bcl-W, Mcl-1, Bfl-1/A1), 2) pro-apoptotic pore-formers (Bax, Bak, Bok) and 3) pro-apoptotic BH3-only proteins (Bad, Bid, Bik, Bim, Bmf, Hrk, Noxa, Puma, *etc*.). All Bcl-2 family proteins contain a BH3 domain; one of four BH domains involves in interactions between these proteins. The anti-apoptotic and pore-forming proteins contain all four BH domains (multi-BH domain proteins) and adopt a highly conserved tertiary structure forming a hydrophobic BH3 domain-binding groove that acts as a receptor for BH3 domains of other family members. The BH3-only proteins are subdivided into activator and sensitizer proteins, containing only the BH3 domain except for Bid are unstructured in solution (10-13). 

**Figure 2:**
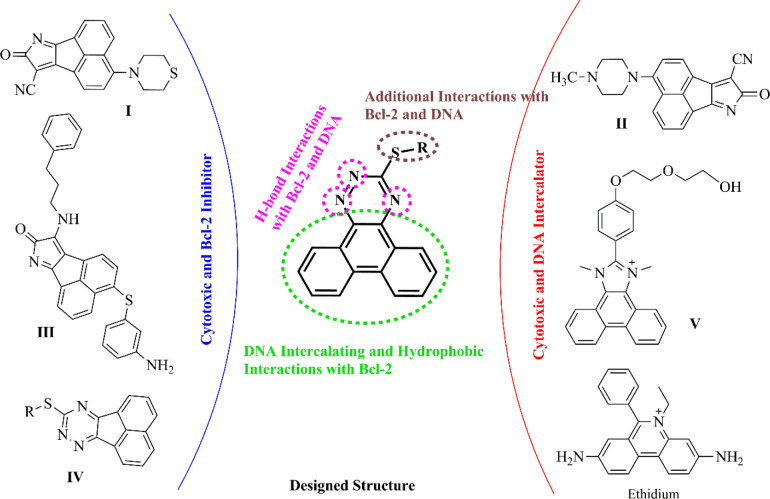
The structural design of novel cytotoxic agents with DNA intercalating and Bcl-2 inhibitory potential based on previously reported scaffolds

1,2,4-Triazine derivatives have attracted great interest owing to their cytotoxic activities against different cancer cells (14, 15). In recent years, some studies on cytotoxic acenaphtho and phenanthrene-based fused scaffolds, as DNA intercalators and Bcl-2 inhibitors, have been performed (Figure 2). Zhang *et al.* reported that acenaphtho-pyrrole derivatives (such as compound **I**) demonstrated cytotoxic effects by DNA intercalation mechanism with an IC_50_ of 10^-7^–10^-8^ M against diverse cancer cell lines and introduced compound **II** as a promising Bcl-2 inhibitor (16-18). Additionally, Song et al. presented compound **III** with an acenaphtho-pyrrole skeleton as a potent pan-Bcl-2-inhibitor (19). Mohammadi *et al*. found out that 9-(alkylthio)-acenaphtho-1,2,4-triazine analogs, **IV**, exhibited moderate to good cytotoxic activities and suggested that these compounds could be potential Bcl-2 inhibitors by molecular docking analysis (20). Wang *et al.* evaluated the cytotoxicity and DNA binding property of some phenanthrene-imidazole derivatives and reported that compound **V** had a high binding affinity with calf thymus DNA (21).

 This studydesigned a novel cytotoxic phenanthro-triazine-3-thiol derivatives with dual activity as DNA intercalators and Bcl-2 inhibitors. After synthesis, the cytotoxicity of the compounds was evaluated against cancer cell lines. Furthermore, to confirm the potential DNA intercalation and Bcl-2 inhibitory activities of phenanthro-triazine scaffolds, molecular docking analysis was performed.

## Methods and materials

*Chemistry*

*Apparatus and reagents*

Melting points were measured on a hot stage apparatus (Electrothermal, Essex, UK) and were uncorrected. ^1^H spectra were recorded on Brucker 500 spectrometer, using TMS as an internal standard, and Chemical shifts (δ) are reported as ppm. IR spectra were obtained on a Perkin-Elmer spectrometer (KBr disk) (Perkin-Elmer, Waltham, MA). Mass spectra were recorded with an Agilent spectrometer (Agilent technologies 9575c inert MSD, USA). All commercially available solvents and reagents were of analytical grade and obtained from Merck and Aldrich.

*Procedure for the synthesis of phenanthro [9,10-e][1,2,4]triazine-3-thiol (P1)*

The phenanthrene-9,10-dione (4.16 g, 20 mmol) was added to 80 mL of acetic acid, and the mixture was heated to about 100 ^o^C with stirring for about half an hour. Thiosemicarbazide (3.65 g, 40 mmol) was added, and the mixture was refluxed for about three hours. The reaction mixture cooled to room temperature and then poured into ice-cold water. The solid product was filtered and washed with cold water. Yield: 67.5%, R_f_ = 0.56 (EtOAc/ PetEther 1:1), mp: 122-124 °C. ^1^H NMR (500 M HZ, CDCl_3_): δ_H_ 14.65 ppm (s, 1H, NH), 8.74-8.39 (m, 2H, phenanthrene-H), 8.19-8.06 (m, 5H, phenanthrene-H), 7.78-7.37 (m, 9H, phenanthrene-H), 6.72 (s, 1H, SH). MS (EI) m/z (%) 263 (38.6), 235 (77.2), 176.1 (100), 150 (10.53). IR (KBr): υ = 3145 (CH-aromatic), 3413 (NH), 3242 cm^-1 ^(SH).

*General procedure for the synthesis of phenanthro[9,10-e][1,2,4]triazine-3-thiol derivatives (P2-P16)*

The phenanthro[9,10-e][1,2,4]triazine-3-thiol (0.263 g, 1 mmol) was added to a stirred hot ethanolic solution of sodium hydroxide (0.1 M). After cooling to room temperature, corresponding alkyl/benzyl halide was added, and the mixture was stirred for about 2-24 h at 25 °C. Completion of the reaction was checked by thin-layer chromatography (TLC). The solvent was reduced under vacuum; the precipitate was filtered and washed with ethanol and cold water. Finally, the dried precipitate was purified by recrystallization or chromatography to give the final pure derivative (Table 1).

*3-(methylthio)phenanthro[9,10-e][1,2,4]tri-azine (P2)*

Compound **P2** was prepared by the described procedure using methyl iodide (3 mmol, 0.19 mL) and the obtained precipitate was recrystallized from ethyl acetate. Yield: 36.1%, R_f_ = 0.67 (EtOAc/ PetEther 1:1), mp: 157-160 °C. ^1^H NMR (500 M HZ, CDCl_3_): δ_H_ 9.36 (d, 1H, *J *= 7.2 Hz, phenanthrene-H), 9.15 (d, 1H, *J *= 7.6 Hz, phenanthrene-H), 8.53 (s, 2H, phenanthrene-H), 7.88-7.72 (m, 4H, phenanthrene-H), 2.89 ppm (s, 3H, CH_3_). MS (EI) m/z (%) 277.1 (43.9), 249.2 (79), 235 (46.5), 203.1 (7), 176.1 (100), 150.1 (14). IR (KBr): υ = 3060 (CH-aromatic), 2927 cm^-1^ (CH-aliphatic).

*3-(ethylthio)phenanthro[9,10-e][1,2,4]tri-azine (P3)*

Compound **P3** was prepared by the described procedure using ethyl iodide (3 mmol, 0.24 mL) and the obtained precipitate was recrystallized from ethyl acetate. Yield: 54.91%, R_f_ = 0.67 (EtOAc/ PetEther 1:1), mp: 131-133 °C. ^1^H NMR (500 M HZ, CDCl_3_): δ_H_ 9.4 (d, 1H, *J = *7.5 Hz, phenanthrene-H), 9.2 (d, 1H, *J = *7.5 Hz, phenanthrene-H), 8.6 (t, 2H, *J = *8 Hz, phenanthrene-H), 7.92-7.74 (m, 4H, phenanthrene-H), 3.5 (q, 2H, *J = *7.2 Hz, CH_2_-CH_3_), 1.6 ppm (t, 3H, *J = *7.2 Hz, CH_2_-CH_3_). MS (EI) m/z (%) 291.1 (40.35), 263.1 (28), 235.1 (100), 203.1 (10), 176.1 (75.44), 150.1 (10.5). IR (KBr): υ = 3065 (CH-aromatic), 2928 cm^-1^ (CH-aliphatic).

*3-(propylthio)phenanthro[9,10-e][1,2,4]tri-azine (P4)*

Compound **P4** was prepared by the described procedure using 1-bromopropane (3 mmol, 0.29 mL), the obtained precipitate was dissolved in appropriate solvent and purified using silica gel column chromatography with petroleum ether-ethyl acetate as the mobile phase. Yield: 49.12%, R_f_ = 0.69 (EtOAc/ PetEther 3:7), mp: 118-120 °C. ^1^H NMR (500 MHZ, CDCl_3_): δ_H_ 9.37 (d, 1H, *J = *7.0 HZ, phenanthrene-H), 9.15 (d, 1H, *J = *7.6 HZ, phenanthrene-H), 8.55 (t, 2H, *J = *7.9 HZ, phenanthrene-H), 7.90-7.30 (m, 4H, phenanthrene-H), 3.48 (t, 2H, *J = *7.2 HZ, CH_2_-CH_2_-CH_3_), 2.0 (sext, 2H, -CH_2_-CH_2_-CH_3_), 1.22 ppm (t, 3H, J = 7.3 HZ, -CH_2_-CH_2_-CH_3_). MS (EI) m/z (%) 305.1 (28), 279.1 (36.9), 263.1 (47.4), 235.1 (100), 203.1 (14), 176.1 (77.2), 150.1 (14). IR (KBr): υ = 3071 (CH-aromatic), 2960, 2929 cm^-1^ (CH-aliphatic). 

*3-(butylthio)phenanthro[9,10-e][1,2,4]tri-azine (P5)*

Compound **P5** was prepared by the described procedure using 1-bromobutane (3 mmol, 0.29 mL), the obtained precipitate was purified using silica gel column chromatography with petroleum ether-ethyl acetate as the mobile phase and then recrystallized from chloroform. Yield: 14%, R_f_ = 0.66 (EtOAc/ PetEther 3:7), mp: 81-83 °C. ^1^H NMR (500 MHZ, CDCl_3_): δ_H_ 9.31 (d, 1H, *J = *7.4 HZ, phenanthrene-H), 9.06 (d, 1H, *J = *7.4 HZ, phenanthrene-H), 8.47 (m, 2H, phenanthrene-H), 7.85-7.67 (m, 4H, phenanthrene-H), 3.48 (t, 2H, *J = *7.3 HZ, CH_2_-(CH2)_2_-CH_3_), 1.96 (pent, 2H, CH_2_-CH_2_-CH_2_-CH_3_), 1.66 (sext, 2H, CH_2_-CH_2_-CH_2_-CH_3_), 1.07 ppm (t, 3H, *J = *7.3 HZ, CH_2_-CH_2_-CH_2_-CH_3_). MS (EI) m/z (%) 319.2 (50.9), 291.1 (19.3), 263.1 (52.6), 235.1 (100), 203.1 (64.9), 176.1 (75.4), 150.1 (14). IR (KBr): υ = 3071 (CH-aromatic), 2963 cm^-1^ (CH-aliphatic).

*3-(isobutylthio)phenanthro[9,10-e][1,2,4]tri-azine (P6)*

Compound **P6** was prepared by the described procedure using 1-bromopropane2-methylpropan (3 mmol, 0.33 ml), the obtained precipitate was purified using silica gel column chromatography with petroleum ether-ethyl acetate as the mobile phase and then recrystallized from chloroform. Yield: 17.8%, R_f_ = 0.67 (EtOAc/ PetEther 2:8), mp: 93-96 °C. ^1^H NMR (500 MHZ, CDCl_3_): δ_H_ 9.4 (d, 1H, *J = *7.9 HZ, phenanthrene-H), 9.2 (d, 1H, *J = *7.9 Hz, phenanthrene-H), 8.6 (t, 2H, *J = *9 HZ, phenanthrene-H), 7.93-7.75 (m, 4H, phenanthrene-H), 3.4 (d, 2H, *J = *6.8 HZ, CH_2_(CH(CH_3_)_2_)), 2.3-2.1 (m , 1H, CH_2_(CH(CH_3_)_2_)), 1.23 ppm (d, 6H, *J = *6.8 Hz, (CH_3_)_2_). MS (EI) m/z (%) 319.2 (10.5), 291.1 (17.5), 263.1 (75.4), 235.1 (100), 203.1 (10.5), 176.1 (49), 150.1 (8.8). IR (KBr): υ = 3060 (CH-aromatic), 2963, 2926 cm^-1^ (CH-aliphatic).

*3-(phenanthro[9,10-e][1,2,4]triazin-3-ylthio)propanenitrile (P7)*

Compound **P7** was prepared by the described procedure using 3-bromopropionitrile (3 mmol, 0.25 mL) and the obtained precipitate was recrystallized from ethyl acetate. Yield: 25.3%, R_f _= 0.27 (EtOAc/ PetEther 3:7), mp: 128-130 °C. ^1^H NMR (500 MHZ, CDCl_3_): δ_H_ 9.4 (d, 1H, *J = *7.8 HZ, phenanthrene-H), 9.17 (d, 1H, *J = *7.8 HZ, phenanthrene-H), 8.62 (t, 2H, *J = *8.6 HZ, phenanthrene-H), 7.93-7.75 (m, 4H, phenanthrene-H), 3.76 (t , 2H, *J = *7.2 HZ, CH_2_-CH_2_-CN), 3.15 ppm (t, 2H, *J = *7.2 HZ, CH_2_-CH_2_-CN). MS (EI) m/z (%) 316.2 (33.3), 288.1 (100), 260.1 (17.5), 235.1 (21.9), 201.1 (4.4), 176.1 (73.7), 150.1 (12.3). IR (KBr): υ = 3064 (CH-aromatic), 2923 (CH-aliphatic), 2245 cm^-1^ (CN).

*3-((2-methoxyethyl)thio)phenanthro[9,10-e][1,2,4]triazine (P8)*

Compound **P8** was prepared by the described procedure using 2-methoxyethyl chloride (3 mmol, 0.29 ml), the obtained precipitate was purified using silica gel column chromatography with petroleum ether-ethyl acetate as the mobile phase and then recrystallized from ethyl acetate. Yield: 16.2%, R_f _= 0.49 (EtOAc/ PetEther 3:7), mp: 100-102 °C. ^1^H NMR (500 MHZ, CDCl_3_): δ_H _9.25 (d, 1H, *J = *7.8 HZ, phenanthrene-H), 9.0 (d, 1H, *J = *7.8 HZ, phenanthrene-H), 8.42-8.39 (m, 2H, phenanthrene-H), 7.82-7.63 (m, 4H, phenanthrene-H), 3.91 (t, 2H, *J = *6.5 Hz, CH_2_-CH_2_-OCH_3_), 3.69 (t, 2H, *J = *6.5 Hz, CH_2_-CH_2_-OCH_3_), 3.5 ppm (s, 3H, OCH_3_). MS (EI) m/z (%) 321 (10.5), 293 (7), 263 (98.3), 23 (100), 203 (73.7), 176 (63.2), 150 (17.5). IR (KBr): υ = 3065 (CH-aromatic), 2962, 2927 cm^-1^ (CH-aliphatic).

*3-(cyclopentylthio)phenanthro[9,10-e][1,2,4]triazine (P9)*

Compound **P9** was prepared by the described procedure using cyclopentyl bromide (3 mmol, 0.32 mL), the obtained precipitate was purified using silica gel column chromatography with petroleum ether-ethyl acetate as the mobile phase and then recrystallized from ethyl acetate. Yield: 11.6%, R_f_ = 0.73 (EtOAc/ PetEther 2:8), mp: 170-172 °C. ^1^H NMR (500 MHZ, CDCl_3_): δ_H_ 9.34 (d, 1H, *J = *7.8 HZ, phenanthrene-H), 9.11 (d, 1H, *J = *8 HZ, phenanthrene-H), 8.50 (t, 2H, *J = *7.7 HZ, phenanthrene-H), 7.87-7.69 (m, 4H, phenanthrene-H), 4.41 (quint, 1H, Hₐ-cyclopenthyl), 2.51-2.46 (m, 2H, H-cyclopenthyl), 1.95-1.82 ppm (m, 6H, H-cyclopenthyl). MS (EI) m/z (%) 331.1 (29.9), 303.1 (22.8), 279.1 (8.8) 262.1 (29.9), 235. 1(100), 208.1 (91.2), 176.1 (63.2), 149 (52.6). IR (KBr): υ = 3032 (CH-aromatic), 2959 cm^-1^ (CH-aliphatic).

*3-(cyclohexylthio)phenanthro[9,10-e][1,2,4]triazine (P10)*

Compound **P10** was prepared by the described procedure using cyclohexyl bromide (3 mmol, 0.37 mL), the obtained precipitate was purified using silica gel column chromatography with petroleum ether-ethyl acetate as the mobile phase and then recrystallized from ethyl acetate. Yield: 15.8%, R_f_ = 0.49 (EtOAc/ PetEther 2:8), mp: 144-147 °C. ^1^H NMR (300 MHZ, CDCl_3_): δH 9.27 (d, 1H, *J = *6 HZ, phenanthrene-H), 9.05 (d, 1H, *J = *6 HZ, phenanthrene-H), 8.45 (t, 2H, *J = *6 HZ, phenanthrene-H), 7.78-7.61 (m, 4H, phenanthrene-H), 4.13 (quint, 1H, Hₐ-cyclohexyl), 1.84-1.80 (m, 2H, H-cyclohexyl), 1.65-1.50 ppm (m, 4H, H-cyclohexyl), 1.37-1.18 ppm (m, 4H, H-cyclohexyl). MS (EI) m/z (%) 345.2 (28), 317.2 (14), 264.1 (31.6), 235.1 (100), 203.1 (17.5), 176.1 (42), 150.1 (7). IR (KBr): υ = 3060 (CH-aromatic), 2962, 2924 cm^-1^ (CH-aliphatic).

*3-(benzylthio)phenanthro[9,10-e][1,2,4]tri-azine (P11)*

Compound **P11** was prepared by the described procedure using benzyl bromide (2 mmol, 0.340 g), the obtained precipitate was purified using silica gel column chromatography with petroleum ether-ethyl acetate as the mobile phase and then recrystallized from ethyl acetate. Yield: 41.4%, R_f_ = 0.65 (EtOAc/PetEther 1:4), mp: 167-170 °C. ^1^H NMR (300 M Hz, CDCl_3_): δ_H_ 9.122 (d, 1H, *J = *5.7 Hz, phenanthrene-H), 8.887 (d, 1H, *J = *7.2 Hz, phenanthrene-H), 8.275-8.245 (m, 2H, Ar-H), 7.655-7.172 (m, 9H, Ar-H), 4.608 ppm (s, 2H, CH_2_ -Phenyl). MS (EI) m/z (%) 353.1 (58.2), 190.1 (100), 91.1 (61.8). IR (KBr): ν 3025 (CH-aromatic), 2963 cm^-1^ (CH-aliphatic).

*3-((4-bromobenzyl)thio)phenanthro[9,10-e][1,2,4]triazine (P12)*

Compound **P12** was prepared by the described procedure using 4-bromobenzyl bromide (2 mmol, 0.500 g), the obtained precipitate was purified using silica gel column chromatography with petroleum ether-ethyl acetate as the mobile phase and then recrystallized from ethyl acetate. Yield: 48.8%, R_f_ = 0.62 (EtOAc/PetEther 1:4), mp: 171-175 °C. ^1^H NMR (300 M HZ, CDCl_3_): δ_H_ 9.304-9.273 (dd, 1H, *J = *7.5, 1.8 Hz, phenanthrene-H), 9.065 (d, 1H, *J = *8.1 Hz, phenanthrene-H), 8.508-8.461 (t, 2H, *J = *7.2 Hz, Ar-H), 7.841-7.627 (m, 4H, Ar-H), 7.389 (s, 4H, Ar-H), 4.598 ppm (s, 2H, CH_2_ -Phenyl). MS (EI) m/z (%) 90.1 (18.3), 190.1 (100), 170.9 (57.9), 433.0 (56.2). IR (KBr): ν 3062 (CH-aromatic), 2960 cm^-1^ (CH-aliphatic).

*3-((4-nitrobenzyl)thio)phenanthro[9,10-e][1,2,4]triazine (P13)*

Compound **P13** was prepared by the described procedure using 4-nitrobenzyl bromide (2 mmol, 0.430 g). The obtained precipitate was purified using silica gel column chromatography with petroleum ether-ethyl acetate as the mobile phase and then recrystallized from ethyl acetate. Yield: 16.6%, R_f_ = 0.46 (EtOAc/PetEther 1:4), mp: 198-202 °C.^1^H NMR (300 M HZ, CDCl_3_): δ_H_ 9.356 (d, 1H, *J = *7.8 Hz, phenanthrene-H), 9.105 (d, 1H, *J = *7.8 Hz, phenanthrene-H), 8.589-8.540 (t, 2H, *J = *7.2 Hz, Ar-H), 8.196 (d, 2H, *J = *8.4 Hz, Ar-H), 7.896-7.702 (m, 6H, Ar-H), 4.787 ppm (s, 2H, CH_2_ -Phenyl). MS (EI) m/z (%) 398.1 (44.3), 322.3 (66.9), 190.0 (100). IR (KBr): ν 3076 (CH-aromatic), 2923 (CH-aliphatic), 1524, 1354 (ArNO_2_), 872 cm^-1^ (C-NO_2_). 

*3-((4-fluorobenzyl)thio)phenanthro[9,10-e][1,2,4]triazine (P14)*

Compound **P14** was prepared by the described procedure using 4-fluorobenzyl chloride (2 mmol, 0.288 g), the obtained precipitate was purified using silica gel column chromatography with petroleum ether-ethyl acetate as the mobile phase and then recrystallized from ethyl acetate. Yield: 61.2%, R_f_ = 0.60 (EtOAc/PetEther 1:4), mp: 157-160 °C. ^1^H NMR (300 M HZ, CDCl_3_): δ_H_ 9.171-9.141 (dd, 1H, J = 7.2, 1.5 Hz, phenanthrene-H), 8.910 (d, 1H, *J = *7.8 Hz, phenanthrene-H), 8.326-8.288 (m, 2H, Ar-H), 7.692-7.438 (m, 6H, Ar-H), 6.976-6.919 (m, 2H, Ar-H), 4.581 ppm (s, 2H, CH_2_ -Phenyl). MS (EI) m/z (%) 371.1 (79.3), 343.1 (35.1), 190.1 (73.9), 109.0 (100) IR (KBr): ν 3070 (CH-aromatic), 2963 cm^-1^ (CH-aliphatic).

*3-((4-methylbenzyl)thio)phenanthro[9,10-e][1,2,4]triazine (P15)*

Compound **P15** was prepared by the described procedure using 4-methylbenzyl chloride (2 mmol, 0.280 g), the obtained precipitate was purified using silica gel column chromatography with petroleum ether-ethyl acetate as the mobile phase and then recrystallized from ethyl acetate. Yield: 34.7%, R_f_ = 0.65 (EtOAc/PetEther 1:4), mp: 138-141 °C. ^1^H NMR (300 M HZ, CDCl_3_): δ_H _9.013-8.990 (m, 1H, phenanthrene-H), 8.766-8.724 (t, 1H, J = 7.2 Hz, phenanthrene-H), 8.112-8.085 (m, 2H, Ar-H), 7.574-7.409 (m, 2H, Ar-H), 4.561 & 4.526 (2s, 2H, CH_2_-Phenyl), 2.227 ppm (s, 3H, CH_3_-Phenyl). MS (EI) m/z (%) 91.1 (32.1), 105.5 (100), 190.1 (96.2), 367.1 (89.1) IR (KBr): ν 3074 (CH-aromatic), 2926 cm^-1^ (CH-aliphatic).

*3-((4-methoxybenzyl)thio)phenanthro[9,10-e][1,2,4]triazine (P16)*

Compound **P16** was prepared by the described procedure using 4-methoxybenzyl chloride (2 mmol, 0.312 g), the obtained precipitate was purified using silica gel column chromatography with petroleum ether-ethyl acetate as the mobile phase and then recrystallized from ethyl acetate. Yield: 31.2%, R_f_ = 0.57 (EtOAc/PetEther 1:4), mp: 152-154 °C. ^1^H NMR (300 M Hz, CDCl_3_): δ_H_ 9.230-9.199 (dd, 1H, *J = *6.9, 1.5 Hz, phenanthrene-H), 9.006-8.975 (dd, 1H, *J = *8.1, 1.2 Hz, phenanthrene-H), 8.371-8.334 (m, 2H, Ar-H), 7.777-7.572 (m, 4H, Ar-H), 7.501-7.472 (d, 2H, *J = *8.7 Hz, ArH), 6.891-6.862 (d, 2H, *J = *8.7 Hz, Ar-H), 4.646 (s, 2H, CH_2_-Phenyl), 3.777 ppm (s, 2H, O-CH_3_). MS (EI) m/z (%) 383.1 (44.8), 190.0 (31.1), 121.0 (100). IR (KBr): ν 3068 (CH-aromatic), 2952 (CH-aliphatic), 1249, 1045 cm^-1^ (C-O-C).

*Biological section*

*Cell lines and cell culture*

This study used two cell lines, including MOLT-4 (human acute lymphoblastic leukemia) and MCF-7 (human breast adenocarcinoma). MOLT-4 was grown in suspension, while MCF-7 was grown in monolayer culture. The MOLT-4 cell line was purchased from the National Cell Bank of Iran, Pasteur Institute, Tehran, Iran, while MCF-7 cells were obtained from the Iranian Biological Resource Center (IBRC), Tehran, Iran. The cells were cultured in RPMI 1640 supplemented with 10% fetal bovine serum (FBS) and 100 units/mL penicillin-G and 100 µg/mL streptomycin at 37 °C in humidified air containing 5% CO_2_. RPMI-1640, FBS, trypsin and phosphate-buffered saline (PBS) were purchased from Biosera (Ringmer, UK). 3-(4,5-Dimethylthiazol- 2-yl)-2,5-diphenyltetrazolium bromide (MTT) was obtained from Sigma-Aldrich (St. Louis, MO, USA). Standard chemotherapeutic agents, dimethylsulfoxide (DMSO) and penicillin/streptomycin were purchased from EBEWE Pharma (Unterach, Netherlands), Merck (Darmstadt, Germany) and Invitrogen (San Diego, CA, USA), respectively. 

*MTT assay*

Cells were seeded into 96-well plates with a density of 5×10^4^ cells/ml (100 µl in each well) and incubated for 24 h at 37 °C. Afterward, the synthesized compounds were added to each well (in triplicates) at three different concentrations. Doxorubicin and cisplatin were used as positive controls. The plates were further incubated for 72 h, and then 80 μL of the medium was removed, and the same volume of an MTT solution was added at a final concentration of 0.5 mg/mL. Plates were incubated for 4 h at 37 °C to allow the formazan crystals to be formed. Then,200 μL DMSO was added to each well to dissolve the formazan crystals. Absorbance was determined at 570 nm with background correction at 655 nm using a Bio-Rad microplate reader (Model 680). The percentage of viability inhibition compared to control was assessed for each concentration of the compound, and IC_50_ values were computed with CurveExpert software version 1.34 for Windows.

*Molecular docking analysis*

Computational docking, an extremely useful tool to achieve deep insight into drug-target interactions, has played a crucial role in drug discovery. The synthesized derivatives were subjected to molecular docking studies using AutoDock Tools and AutoDock software in this study.

*Molecular docking studies with DNA*

The X-ray crystal structure of B-DNA (PDB ID: 1Z3F) was obtained from the Protein Data Bank. The ligand structures were drawn and minimized under Semi-empirical AM1 methods using HyperChem 7. Molecular docking studies were done using the AutoDock Tools 1.5.7rc1 and AutoDock 4.2.5.1 docking programs. The energy calculations were made using genetic algorithms (LGA) with the number of GA runs set to 100. 

*Molecular docking studies with Bcl-2 protein*

The crystal structure of Bcl-2 (PDB ID: 3ZLN) was acquired from RCSB Protein Data Bank (http://www.rcsb.org).Innate ligand and waters were removed, hydrogen atoms were added, non-polar hydrogens were merged, and Gasteiger charges were added using AutoDock Tools to prepare the protein for docking. 3D structures of ligands were sketched and minimized under Molecular Mechanics MM+ and then Semi-empirical AM1 methods using HyperChem software. A grid of 60, 60, and 60 points in x = -17.788, y = -12.682 and z = 10.891 directions with 0.375 Å grid spacing was built. The docking process was performed by AutoDock 4.2. To find the conformers with the lowest binding energy, Lamarckian genetic algorithm (LGA) was used, and the number of GA runs was set to 100. The other parameters were left at program default values. Docking procedure validity was examined by re-docking the structure co-crystallized ligand into the receptor (self-docking) using the protocol mentioned above.

## Results and Discussion

*Design strategy*

The target compounds were designed by a molecular hybridization strategy based on the structures of some DNA intercalators and Bcl-2 inhibitors (**I**-**V** and ethidium illustrated in Figure 2) previously reported in the literature. As shown in Figure 2, the molecular hybridization of phenanthrene and 1,2,4-triazine rings yielded the scaffold which contains three parts: 1) a phenanthrene scaffold as a planar polycyclic central core to intercalate between DNA base-pairs and to provide π-π interactions with the key amino acids in the active site of Bcl-2, 2) a 1,2,4-triazine ring which is fused to phenanthrene as a site to establish H-bond interactions with DNA nucleic acids and Bcl-2 target and 3) a variety of benzyl-thio and alkyl-thio side chains to improve the interactions and promote the physicochemical properties. To the best of our knowledge, there have been no literature reports regarding fused phenanthrene-triazine skeleton bearing alkyl/benzyl-thio substitutions as cytotoxic agents with potential dual DNA intercalating and Bcl-2 inhibitory activity so far. 

*Synthesis*

**Scheme 1:**
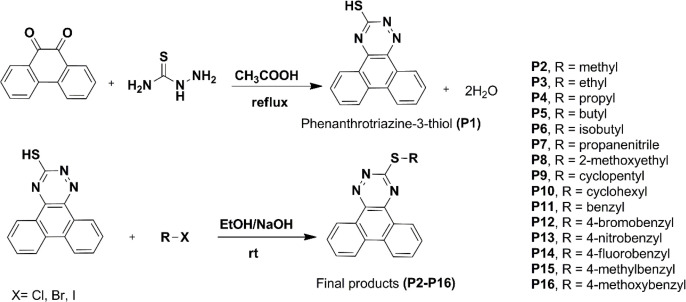
Synthetic approaches to compounds P1-P16

The synthetic routes of **P1-P16 **compounds are outlined in Scheme 1 and Table 1. The reaction of phenanthrene-9,10-dione in refluxing acetic acid with thiosemicarbazide afforded phenanthrotriazine-3-thiol (**P1**). Substitution reaction of different halides with intermediate **P1** in an ethanolic solution of sodium hydroxide furnished phenanthro-triazine-3-thiol derivatives (**P2-P16**). The chemical structures were characterized by IR, MS, ^1^H NMR analysis.

*Cytotoxicity*

The compounds were tested for their *in-vitro* cytotoxicity against MOLT-4 (human acute lymphoblastic leukemia) and MCF-7 (human breast adenocarcinoma) cell lines using the MTT assay. MOLT-4 and MCF-7 are frequently used cell lines for screening anticancer agents included in the NCI-60 panel of cell lines suggested by the National Cancer Institute for *in-vitro* tests. In addition, these cell lines express easily detectable levels of Bcl-2 and Bcl-xl and hence are valuable tools for the study of the inhibitors of these targets (22-24). The chemotherapeutic drugs doxorubicin and cisplatin were used as the positive controls. The results of the MTT assay expressed as IC_50_ values of synthesized compounds are listed in Table 2. The phenanthro-triazine-3-thiol derivative** P1** exhibited considerable cytotoxicity against MOLT-4 with an IC_50_ value of 7.1 ± 1.1 μM and also showed a moderate effect against MCF-7 cells with an IC_50_ value of 26.6 ± 1.6 μM. **P11** (bearing phenyl substitution) demonstrated good cytotoxic activities against MCF-7 with IC_50 _values of 15.4 ± 2.9, while **P15** (bearing 4-methylphenyl substitution) had an IC_50_ of 19.4 ± 2.5 μM against MOLT-4 cells. Compounds **P7**, **P8** and **P14**, containing propanenitrile, 2-methoxyethyl, and 4-fluorophenyl moieties, respectively, exhibited moderate cytotoxic activities against MCF-7 cells with IC_50_ values in the range of 34.3-39.7 μM.

*Molecular docking studies*

*Molecular docking studies with DNA duplex with the* *sequence of d* *(CGATCG)*_2_

**Figure 3:**
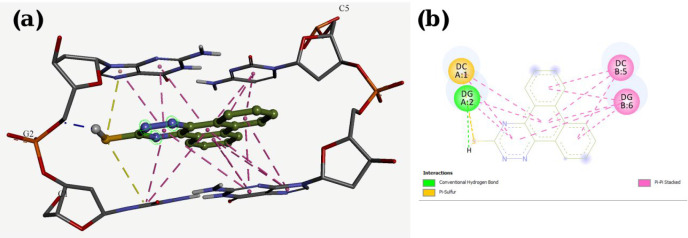
Docking result and interactions of compound **P1** with DNA base pairs in (a) 3D and (b) 2D representation. (G: deoxyguanine, C: deoxycytidin).

**Figure 4:**
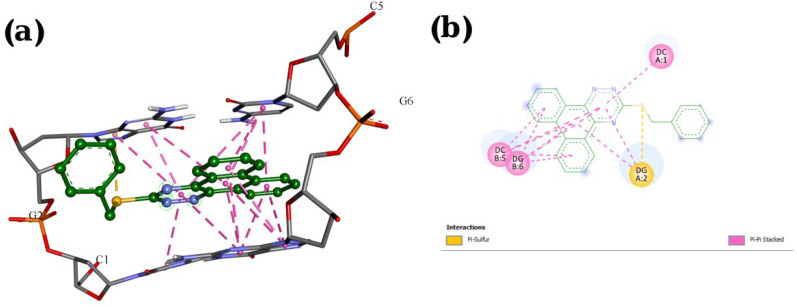
Docking result and interactions of compound **P11** with DNA base pairs in (a) 3D and (b) 2D representation. (G: deoxyguanine, C: deoxycytidin)

**Figure 5:**
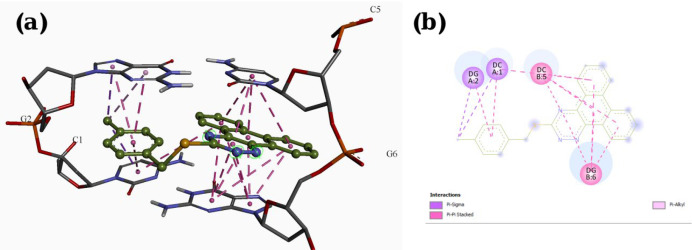
Docking result and interactions of compound **P15 **with DNA base pairs in (a) 3D and (b) 2D representation. (G: deoxyguanine, C: deoxycytidin)

**Figure 6:**
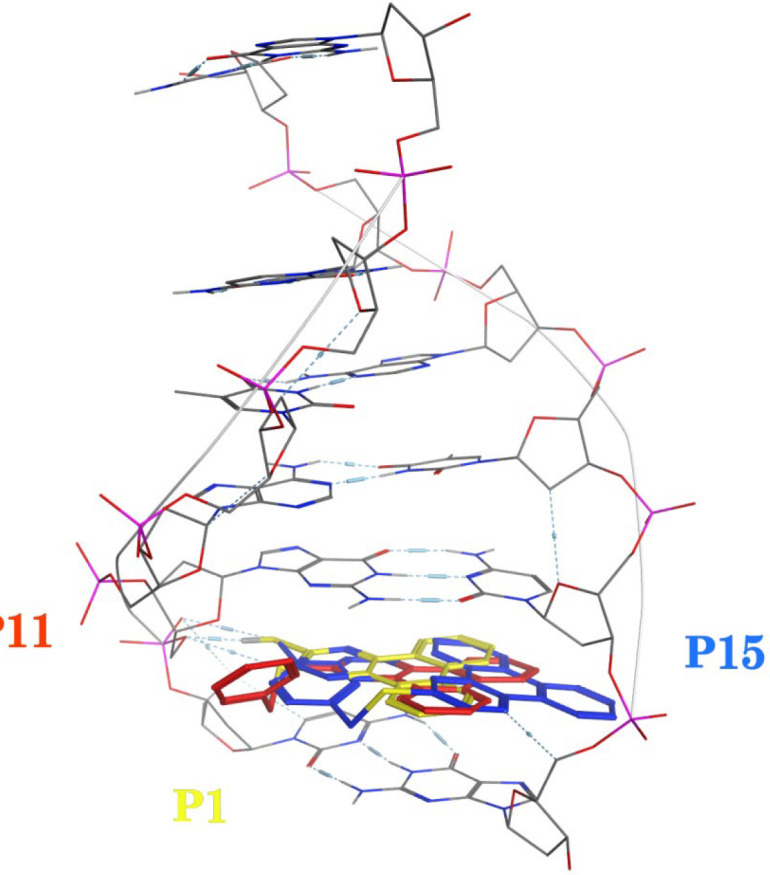
Dock poses of compounds **P1** (yellow), **P11** (red) and **P15** (blue) with DNA

A molecular docking study was employed to identify the binding interactions and estimate the binding affinity of the most active derivatives to DNA duplex with sequence d(CGATCG)_2_. 3D representations of docking results of compounds **P1**, **P11** and **P15** with DNA are depicted in Figures 3-5, and the dock poses of the compounds are illustrated in Figure 6. Considering the values of free energies of binding mentioned in Table 3, it could be deduced that the compounds were properly accommodated between the DNA base pairs. Moreover, as shown in Figures 3-6, the ligands unwound DNA by establishing some interactions with the GC base pairs. The molecular docking analysis revealed that in all three derivatives, the phenantrene-triazine core has contributed in hydrophobic and Pi-Pi interactions with the base pairs. Additionally, in **P1,** the hydrogen atom attached to the sulfur atom provided a hydrogen bond interaction with deoxyguanosine 6 ( DG6) while **P15**, 4-methylphenyl substitution established an additional Pi-Pi interaction with deoxycytidine 5 (DC5).

*Molecular docking studies with Bcl-2 protein*

**Figure 7:**
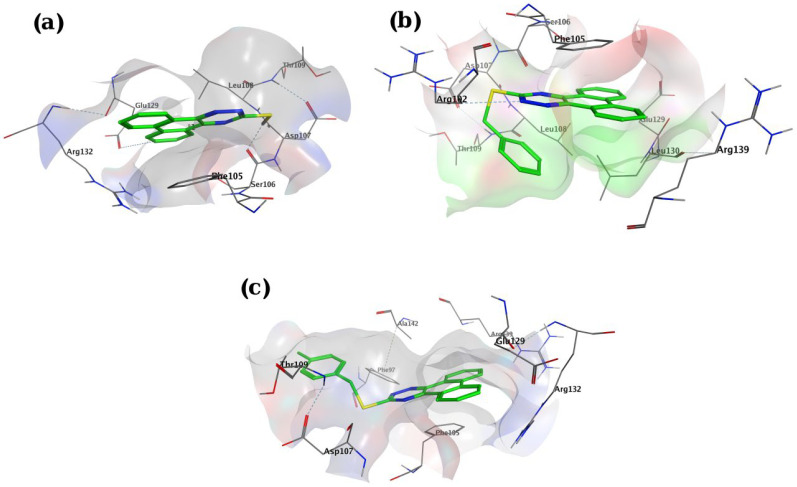
View of binding pocket of Bcl-2 docked with compounds **P1** (a), **P11** (b) and **P15** (c). Bcl-2 is shown as surface, and for ligands carbon, nitrogen and sulfor atoms are shown in green, blue and yellow, respectively

**Figure 8:**
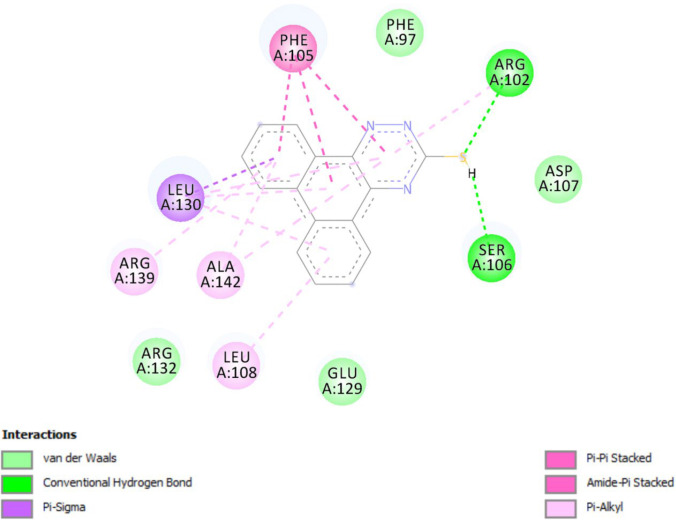
Docking result of compound **P1** in the active site of Bcl-2. Hydrogen bonding, Pi-sigma, Pi-Pi stacked and Pi-alkyl interactions are shown in green, violent, pink and pale pink, respectively

**Figure 9:**
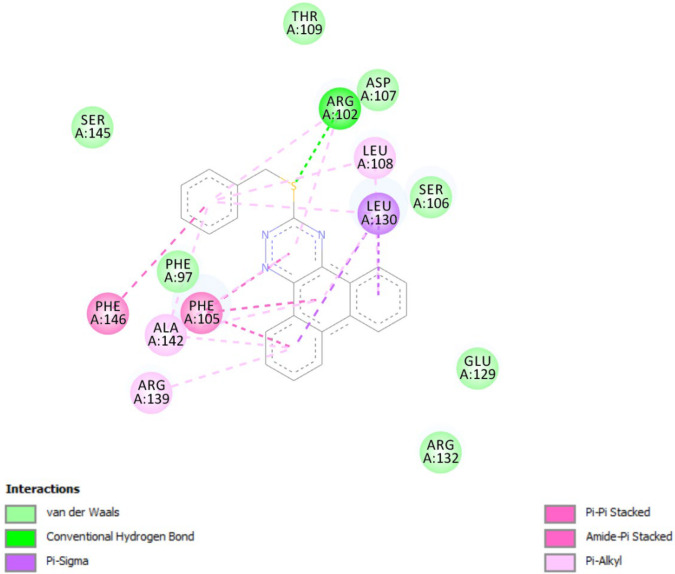
Docking result of compound **P11** in the active site of Bcl-2. Hydrogen bonding, Pi-sigma, Pi-Pi stacked and Pi-alkyl interactions are shown in green, violent, pink and pale pink, respectively

**Figure 10:**
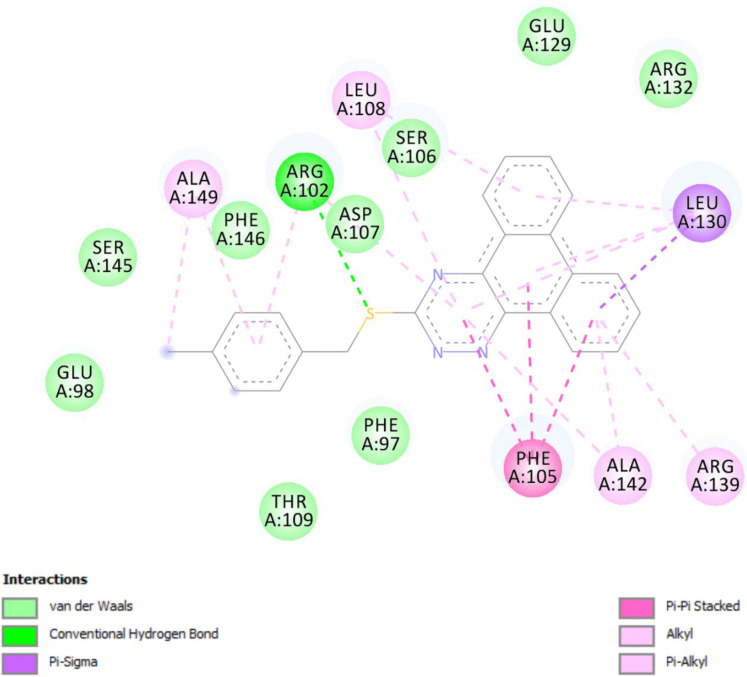
Docking result of compound **P15** in the active site of Bcl-2. Hydrogen bonding, Pi-sigma, Pi-Pi stacked and Pi-alkyl interactions are shown in green, violent, pink and pale pink, respectively

**Figure 11:**
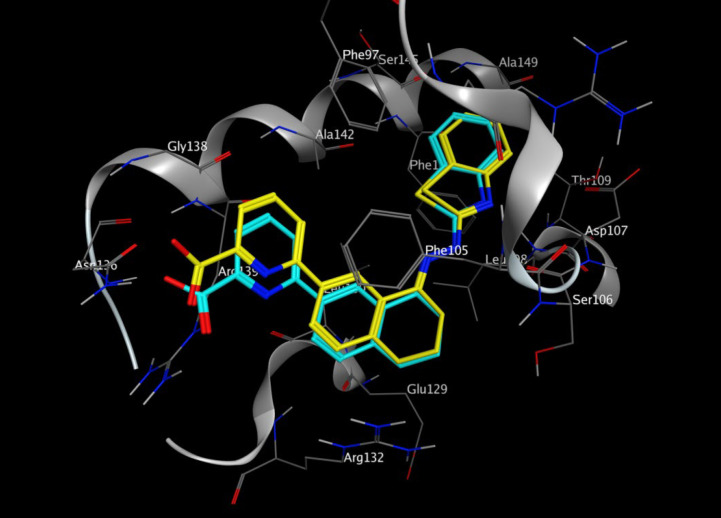
Representation of the co-crystallized inhibitor (cyan) docked into the binding site and superimposed on the cognate ligand (yellow) in the crystal structure of Bcl-2 (PDB ID: 3ZLN).

*In-silico* molecular docking was performed to gain information on the binding modes of the most active compounds (**P1**, **P11,** and **P15**) in the Bcl-2 active site. All the three phenanthro-triazine-3-thiol derivatives were well accommodated into the active site of Bcl-2 (as depicted in Figure 7) and showed good binding energies ranging from -9.96 to -12.58 kcal/mol. The Bcl-2 residues proposed to interact with the ligand molecules are represented in Figures 8-10. The corresponding free energies of binding and the molecular interactions are summarized in Table 4. Molecular docking analysis revealed that the derivatives might inhibit Bcl-2 protein mainly *via* Pi interactions. The tow types of Pi interaction including Pi-alkyl and Pi-Pi stacking as well as hydrogen bonding mainly govern the binding modes of phenanthro-triazine-3-thiol derivatives in the binding site of the protein. In the case of **P1,** two hydrogen bonds were observed between the hydrogen attached to sulfur and Ser106 and Arg102 residues. Moreover, the **P1** derivative interacts with residues Leu108, Arg102, Arg139, Ala142, Leu130, and especially Phe105 through Pi-alkyl, Pi-Pi stacking and Pi-sigma with its triazine and phenanthrene moieties. The same interactions occured in the **P11** and **P15** derivatives. By comparing the residues which are responsible to free energy binding of three derivatives, it is understandable that the phenyl moiety in the **P11** derivative interacting with Leu108, Arg102, Leu130 and Phe146 through Pi-alkyl and Pi-Pi stacking interactions can make a drastic impact on the binding energy, ΔG, of that ligand (-8.86 *versus* -10.98 kcal/mol). It is also referred that the same trend is predicted for **P15** by forming Pi-Pi stacking, Pi-alkyl and alkyl-alkyl interactions between its methylphenyl moiety and Phe146, Leu130 and Ala149 which lead to better binding free energy (-8.86 *versus* -11.73 kcal/mol). By analyzing the contributed residues, it can be concluded that hydrophobic residues primarily contribute to the binding of three derivatives with Bcl-2. Result for re-docking the co-crystallized ligand into the receptor (self-docking) is depicted in Figure 11 and the interactions are listed in Table 4. The root mean square deviation (RMSD) between the best pose of co-crystallized ligand docked into the binding site and the one in the crystal structure was 0.61 Å. In the case of the innate ligand, hydrogen bonding and charg-charg interactions played the key role, as the carboxylate moiety established strong interactions with Asn136 and Arg139 residues. İn addition, benzothiazol and hydrazin moieties contributed in forming two other hydrogen bonds with Leu108 and Ser106. Resıdues Ala149, Phe146, Leu108, Leu130, Phe105 and Arg139 involved in hydrophobic interactions, as seen in docking of compounds **P1**, **P11** and **P15**.

**Table 1:** Chemical structure of synthesized compounds **P1-P16**

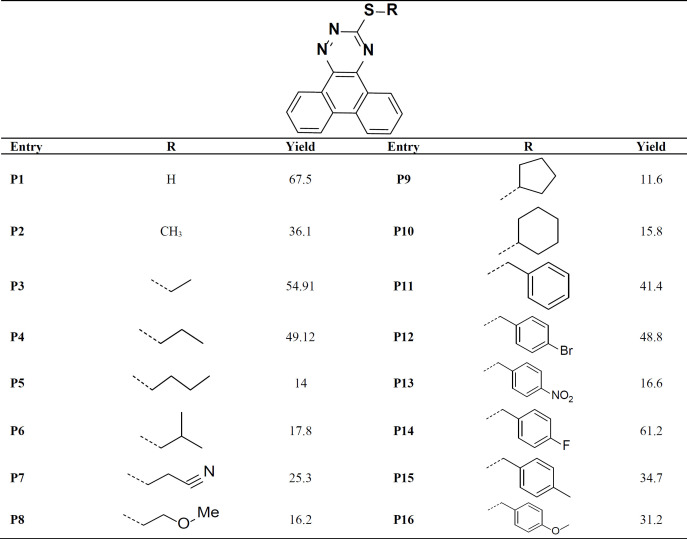

**Table 2:** Cytotoxic activity of synthesized compounds against human cancer cell lines evaluated by MTT reduction assay

**Compound**	**IC**_50_ ** (μM) ± SEM** ^a^			**IC**_50_ ** (μM) ± SEM**	
	**MOLT-4**	**MCF-7**	**Compound**	**MOLT-4**	**MCF-7**
**P1**	7.1 ± 1.1	26.6 ± 1.6	**P10**	˃ 50	˃ 50
**P2**	˃ 25	˃ 10	**P11**	˃ 25	15.4 ± 2.9
**P3**	˃ 10	˃ 10	**P12**	˃ 10	˃ 10
**P4**	˃ 100	˃ 100	**P13**	˃ 50	˃ 50
**P5**	˃ 100	˃ 100	**P14**	25	34.3 ± 2.1
**P6**	˃ 50	˃ 50	**P15**	19.4 ± 2.5	˃ 25
**P7**	˃ 50	39.7 ± 6.1	**P16**	˃ 25	˃ 25
**P8**	˃ 50	33.5 ± 8.8	**Doxorubicin**	0.016 ± 0.004	0.050 ± 0.008
**P9**	˃ 25	˃ 100	**Cisplatin**	3.6 ± 0.6	9.5 ± 1.4

^a^IC_50_: Concentration required for 50% inhibition of tumor cell growth. Values represent the mean of 3–5 experiments ± SEM.

**Table 3:** Docking results of compounds with DNA (PDB ID: 1Z3F).

**Distance (Å)**	**Nucleic acid**	**Moiety of ligand**	**Interactions**	**Ki** **(nM)**	**ΔG**_ (_ **kcal/mol)**	**Compound**
	DC1, DC2DG5, DG6	Phenanthrene S H-S	π -π stacking π –Sulfur H-bond	152.16	-9.19	**P1**
	DC1, DC2DG5, DG6	Phenanthrene	π -π stacking	118.64	-9.45	**P2**
	DC1, DC2DG5, DG6	Phenanthrene	π -π stacking	105.14	-9.52	**P3**
3.066	DC1, DC2DG5, DG6	S Phenanthrene	H-bond, π -π stacking	120.09	-9.44	**P4**
	DC1, DC2DG5, DG6	Phenanthrene	π -π stacking	71.09	-9.75	**P5**
	DC1, DC2DG5, DG6	Phenanthrene	π -π stacking	68.85	-9.77	**P6**
2.760	DC1, DC2DG5, DG6	S Phenanthrene	H-bond, π -π stacking	86.47	-9.64	**P7**
2.775 2.939	DC1, DC2DG5, DG6	O Phenanthrene	H-bond, π -π stacking	110.88	-9.49	**P8**
	DC1, DC2DG5, DG6	Phenanthrene	π –π stacking	20.23	-10.50	**P9**
2.760	DC1, DC2DG5, DG6, DA3	N Phenanthrene	H-bond, π -π stacking	25.23	-10.37	**P10**
	DC1, DC2DG2, DG6	Phenanthrene S	π –π stacking π - Sulfur	94.52	-9.58	**P11**
	DC1, DC2DG5, DG6, DA3	Phenanthrene	π –π stacking	9.11	-10.97	**P12**
	DC1, DC2DG5, DG6, DA3	Phenanthrene	π –π stacking	52.36	-9.93	**P13**
	DC1, DC2DG5, DG6	Phenanthrene	π –π stacking	23.47	-10.41	**P14**
	DC1, DC2DG5, DG6	Phenanthrene Phenyl CH_3_-Phenyl	π –π stacking π – Sigma π --Alkyl	11.61	-10.83	**P15**
3.127	DC1, DC2DG5, DG6, DA3	S Phenanthrene	H-bond, π -π stacking	21.89	-10.45	**P16**

**Table 4.** Interaction data of the most potent cytotoxic derivatives and the cocrystallized ligand (6-[(8E)-8-(1,3-benzothiazol-2-ylhydrazinylidene)-6,7-dihydro-5H-naphthalen-2-yl]pyridine-2-carboxylic acid) with Bcl-2 protein (PDB ID: 3ZLN).

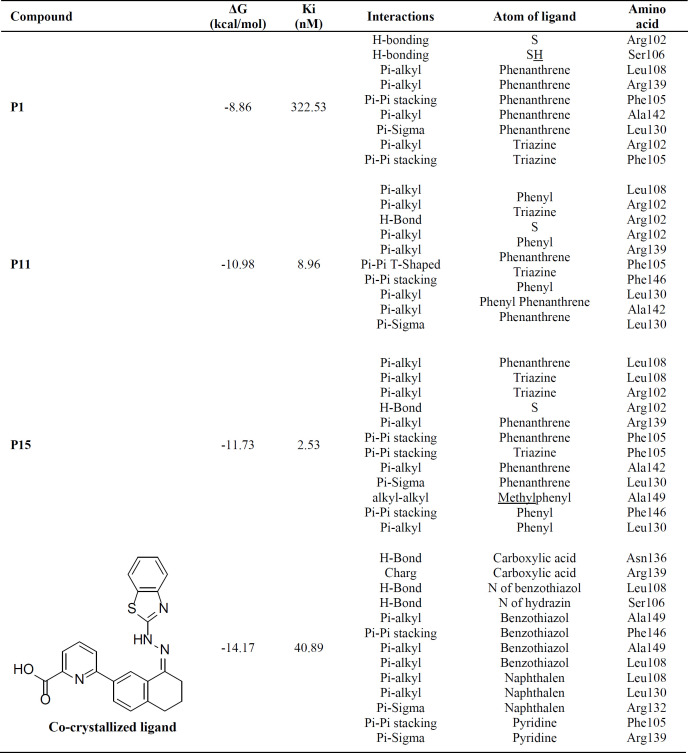

## Conclusion

Phenanthro-triazine-3-thiol derivatives have been synthesized and evaluated for their cytotoxic activity against MOLT-4 and MCF-7 cancer cell lines. Compounds **P1**, **P11** and **P15** were found to be the most potent cytotoxic agents. The docking study revealed that these derivatives unwounded the DNA double helix mainly via hydrophobic interactions with the GC base pairs. Additionally, the active compounds were well accumulated in the active site of Bcl-2 through interactions with Leu108, Ser106, Phe105, Ala142, Leu30, Ala149 and Phe146. Therefore, **P1**, **P11,** and **P15** could be proposed as effective cytotoxic agents with potential DNA intercalation and Bcl-2 inhibitory activities. Further biological evaluations are necessary to confirm our findings.
